# Experimental Methods to Study the Pathogenesis of Human Enteric RNA Viruses

**DOI:** 10.3390/v13060975

**Published:** 2021-05-25

**Authors:** Somya Aggarwal, Ebrahim Hassan, Megan T. Baldridge

**Affiliations:** 1 Division of Infectious Diseases, Department of Medicine, Edison Family Center for Genome Sciences & Systems Biology, Washington University School of Medicine, St. Louis, MO 63110, USA; s.aggarwal@wustl.edu (S.A.); e.hassan@wustl.edu (E.H.); 2 Department of Molecular Microbiology, Washington University School of Medicine, St. Louis, MO 63110, USA

**Keywords:** human norovirus, human rotavirus, human astrovirus, gastroenteritis, immortalized cell lines, human enteroids

## Abstract

Every year, millions of children are infected with viruses that target the gastrointestinal tract, causing acute gastroenteritis and diarrheal illness. Indeed, approximately 700 million episodes of diarrhea occur in children under five annually, with RNA viruses norovirus, rotavirus, and astrovirus serving as major causative pathogens. Numerous methodological advancements in recent years, including the establishment of novel cultivation systems using enteroids as well as the development of murine and other animal models of infection, have helped provide insight into many features of viral pathogenesis. However, many aspects of enteric viral infections remain elusive, demanding further study. Here, we describe the different in vitro and in vivo tools available to explore different pathophysiological attributes of human enteric RNA viruses, highlighting their advantages and limitations depending upon the question being explored. In addition, we discuss key areas and opportunities that would benefit from further methodological progress.

## 1. Introduction

Acute gastroenteritis, characterized by symptoms including nausea, vomiting, malaise, abdominal pain, fever, and diarrhea, is one of the most common health problems worldwide. More than 700 million cases occur annually in children under five years of age, resulting in few deaths in developed countries, but more than 2 million deaths in developing countries [1]. A diverse group of viral, bacterial, and parasitic pathogens are responsible for acute gastroenteritis, but among these, enteric viruses cause almost half of the cases affecting patients of all ages worldwide, and in the United States, viruses are the leading cause [2]. Viral gastroenteritis is usually self-limiting with symptom resolution occurring within a few days, but illness can be prolonged in immunocompromised individuals [3]. Unlike bacterial or parasitic pathogens, enteric viruses cannot be treated with antibiotics, and vaccines are not currently available for many of the key drivers of gastroenteritis.

Examination of intestinal contents during diarrheal illness by electron microscopy resulted in the discovery of numerous viral enteropathogens, now classified as caliciviruses, rotaviruses, astroviruses, or ‘enteric’ adenoviruses [1]. Among these, rotaviruses were the single most important cause of life-threatening diarrhea in children less than five years of age, with an estimated 453,000 pediatric deaths annually in developing countries [4]. However, with the global introduction of the rotavirus vaccine, noroviruses are now recognized as the most important cause worldwide of outbreaks of viral gastroenteritis in humans of all age groups [5,6]. Following rotavirus and norovirus, astroviruses and sapoviruses are the leading viral causes of sporadic gastroenteritis in children [7,8]. Other viruses associated with gastroenteritis in humans include coronaviruses, toroviruses, picornaviruses, and picobirnaviruses [1]. With progressive advances in sequencing-based approaches to identify causative pathogens, we are increasingly adept at defining those viruses causing symptoms, characterizing their prevalence during asymptomatic infection, and identifying novel viral pathogens [9,10]. However, characterization of the effects of established or novel strains on the host requires the use of both in vitro and in vivo experimental model systems, many of which have, until recently, been lacking for these pathogens.

The past decade has witnessed the advent of intestinal organoid-based studies of virus–host interactions as well as an expansion in available animal models, both of which have dramatically increased our capacity to decipher physiologically relevant viral and cellular processes during infection. Here, we discuss the in vitro and in vivo tools that are currently used to study the pathogenesis of human RNA enteric viruses, focusing on norovirus, rotavirus, and astrovirus, to provide a useful compendium for those beginning to explore the field of gastroenteritis (Table 1 and Table 2).

## 2. Human RNA Enteric Viruses

Human RNA enteric viruses include an array of viral genera that are predominantly transmitted through the fecal-oral route and can invade and replicate in the gastrointestinal tract. These viruses can be categorized as: (1) viruses predominantly infecting the small intestinal epithelium to cause acute gastroenteritis, such as rotaviruses, caliciviruses, and astroviruses; (2) viruses that briefly infect the gastrointestinal tract before causing clinical disease at extraintestinal sites, such as polioviruses, coxsackieviruses, and hepatitis A (HAV) and hepatitis E (HEV); and (3) viruses that infect the intestinal tract at late stages of disease in immunocompromised hosts such as human immunodeficiency virus. In this review, we will predominantly focus on this first category of viruses that infect the intestinal epithelium, and here will briefly describe what is known about their pathogenesis and cellular tropism.

### 2.1. Rotavirus

Human rotavirus (HRV) is a non-enveloped, segmented, double-stranded RNA virus from the family Reoviridae, first identified from duodenal biopsies and fecal samples of children suffering from diarrhea [48,49]. A leading cause of acute gastroenteritis in children under five, HRV infection can range from asymptomatic to causing severe non-bloody diarrhea with vomiting and fever lasting from 3 to 8 days [50,51,52,53]. In some cases, rapid dehydration and electrolyte imbalance can lead to death [54,55], and indeed prior to the development of effective vaccines, HRV claimed more than 500,000 lives each year and accounted for an estimated $1 billion in health care costs in the US annually [4,56]. RV can be classified into seven major serogroups (A–G). Groups A, B, and C infect both humans and animals, while the rest have only been found in animals to date, and Group A has been established as the most common RV responsible for causing human illness [57]. These viruses possess a distinct cell tropism, predominantly infecting the mature enterocytes and enteroendocrine cells of the small intestine [19,58]. It has been recently observed that transmission of HRV can occur via vesicle-cloaked virus clusters, indicating that both free and clustered virions may contribute to disease pathogenesis [59].

HRV antigen can be detected in stool samples using ELISA or immunochromatography, but qPCR-based assays provide greater sensitivity and allow genotyping of virus isolates, and thus are routinely used in vaccine and epidemiological studies [60,61]. Currently, two live, attenuated RV vaccines are widely used worldwide: the RV5 or RotaTeq vaccine is a pentavalent vaccine composed of five bovine-human RV strains [62], while the RV1 or Rotarix vaccine is monovalent containing one HRV strain [63]. Since the introduction of these vaccines in 2006 in national vaccination programs of America and Europe [64], a massive reduction in the incidence of HRV infection has occurred [63,65,66]. Based on the performance of these vaccines, in 2009, the WHO extended this recommendation worldwide. Although the effectiveness of RV vaccines remains higher in developed countries than in developing countries, potentially due to a variety of factors including microbiota variation, implementation of these vaccines has universally translated into a substantial reduction in the incidence of severe RV infection [67,68,69].

### 2.2. Norovirus

Human noroviruses (HuNoVs) are non-enveloped, positive-sense RNA viruses in the family Caliciviridae [70], and are the leading cause of acute gastroenteritis worldwide. Colloquially referred to as the ‘winter vomiting bug’, HuNoV was first identified in stool samples collected from a diarrheal outbreak in Norwalk, Ohio and thus the original strain was called ‘Norwalk virus’ [71]. In developed countries with RV vaccine programs, HuNoV surpasses HRV as the most common cause of gastroenteritis in children. Common symptoms of infection include nausea, vomiting, and diarrhea, usually resolving in 1–2 days, but HuNoV infection can also be asymptomatic. Previous human challenge studies indicate that approximately 30% of HuNoV-infected individuals exhibit no symptoms despite high levels of viral shedding [72,73]. Based on amino acid homology of the major viral capsid protein VP1, the NoV genus is divided into ten genogroups (GI-GX) which are further sub-divided into different genotypes containing individual virus strains [74]. Among these, GI, GII and GIV strains infect humans and genotype 4 of GII (GII.4) is responsible for the majority of human outbreaks [75]. NoVs are generally species-specific, with each genogroup of NoV infecting distinct groups of hosts [76]. While the cellular tropism for HuNoV has been a subject of some controversy, the development of in vitro culture systems, discussed in detail below, have suggested mature enterocytes and B cells as targets for HuNoV [29,31,77]. A dual tropism for immune cells and intestinal epithelial cells, specifically enteroendocrine cells, is supported by histologic analyses of intestinal tissues from HuNoV-infected immunocompromised patients [78,79]. HuNoV clusters cloaked within vesicles have, as for HRV, been implicated in viral transmission [59].

Diagnosis of HuNoV can be performed using commercially available enzyme immunoassays or immunochromatographic assays on samples including diarrheal stool or vomitus. Multiple real-time qRT-PCR (qPCR) assays have also been developed for standard diagnostic use [80]. While there are no HuNoV vaccines currently available, multiple vaccine candidates designed to target the viral capsid have been evaluated in clinical trials [81].

### 2.3. Astrovirus

Human astroviruses (HAstVs) were first reported in 1975, after electron microscopic analysis of stool following an outbreak of pediatric diarrhea revealed virions with a characteristic star-like appearance [82,83]. HAstVs are non-enveloped, positive-sense RNA viruses from the family Astroviridae which have been classified into three divergent groups: the classic HAstVs, the nonclassic HAstV-MLB and HAstV-VA/HMO groups [7]. Among these, classic HAstVs include eight serotypes and are responsible for 2–9% of all acute viral gastroenteritis in children worldwide [7]. The more recently discovered nonclassic AstVs are less well-studied, and their pathological effects and prevalence worldwide remain unclear. In general, HAstV induces diarrheal disease that is milder than HRV or HuNoV, and is associated with abdominal pain, vomiting, and fever that lasts 2–3 days. Although infections are generally self-limiting, immunocompromised individuals may succumb to disseminated infection [84,85]. Asymptomatic infections have been reported in children as well as adults [86,87]. HAstV antigen has been found in the mature enterocytes of the small intestine [88,89,90], and an in vitro cultivation system in human intestinal enteroids supports this tropism, but also identifies goblet cells and intestinal progenitor cells as potentially permissive to infection [43]. No vaccines are currently available against HAstVs, and dehydration secondary to gastroenteritis is treated with oral or intravenous fluids. Currently, real-time RT-quantitative PCR assays are sensitive, fast, and reproducible for HAstV diagnosis [91], and next-generation sequencing continues to facilitate the identification of new emerging strains [92,93].

## 3. In Vitro Tools to Study Human Enteric RNA Viruses

**Figure 1:**
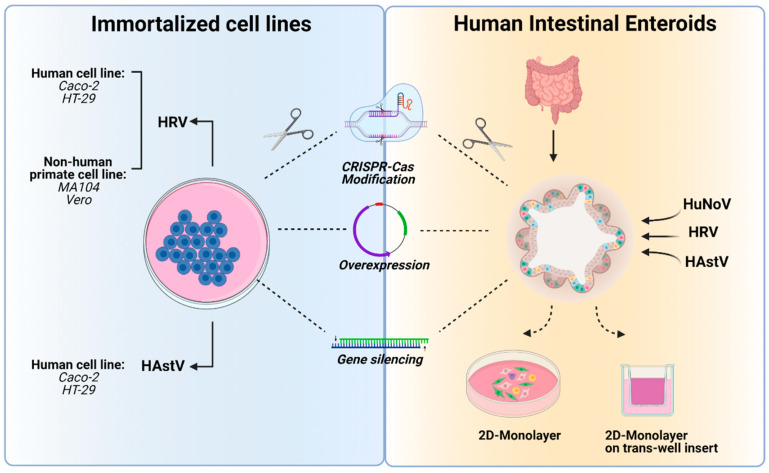
In vitro methods commonly used for the study of human noroviruses (HuNoVs), rotaviruses (HRVs) and astroviruses (HAstVs). Immortalized cell lines and human intestinal enteroids have been used for the study of human enteric viruses. Modifications to cells either to facilitate infections and/or interrogate the role of host genes during infection can be performed using methods including CRISPR-Cas9-based editing, gene overexpression or silencing. Created with BioRender.com (accessed on 21 January 2021).

Although composed of only a single cell layer, the human intestinal epithelium is comprised of a variety of cell types including enterocytes, goblet cells, Paneth cells, enteroendocrine cells, and stem cells [94]. Enterocytes are critical for their absorptive capacity, while secretory cells such as goblet and Paneth cells play an important role in maintaining the epithelial barrier through secretion of mucus and antimicrobial peptides, preventing microbial encroachment from the lumen [95]. Culturing of human enteric viruses in vitro has often proven to be a challenge due to the complexity of the human intestinal epithelium [96], but multiple methods have been developed to study viral interactions with host epithelial cells. Immortalized cell lines have often been a first choice, with many advantages including that they are accessible, inexpensive, scalable, stable, and easy to maintain; these have proven tractable for HRV and HAstV, though a reliable and reproducible in vitro system for HuNoV has been a greater challenge. With the development of human enteroid systems, however, cultivation of nearly any virus with an epithelial cell tropism appears practical. An important consideration for in vitro viral experimentation, applicable to enteric RNA viruses [97,98,99,100], is the potential for passaged viruses to mutate substantially as they adapt to in vitro conditions. While fundamental aspects of viral pathogenesis may remain intact even with viral genetic changes, interpretation of results should always be considered with this caveat in mind. Here, we will discuss the in vitro systems available for studying human RNA enteric viruses (Figure 1).

### 3.1. Immortalized Cell Lines

Immortalized cell lines provide a pure population of cells that are easy to maintain and have the potential to divide indefinitely, and thus many host–enteric virus interaction studies have been performed using cancer-derived or immortalized cell lines. Different human intestinal cell lines, manifesting specific functions and characteristics of the gut epithelium, are widely used, and African green monkey kidney cell lines, which are more susceptible to a variety of viruses, have also served as useful lines for enteric viral research [101].

#### 3.1.1. Adenocarcinoma Cell Lines

Cancer-derived cell lines from different parts of the human intestine are commercially available and have been widely used to explore gut–pathogen interactions, with Caco-2 and HT-29 serving as two predominant colorectal adenocarcinoma cell lines employed.

Caco-2 cells form a polarized monolayer that can differentiate into cells with a remarkable resemblance to enterocytes in the intestinal epithelium [102,103], and they have been successfully employed as an in vitro amplification system for a variety of human enteric viruses [104]. They can be grown as 3-dimensional Transwell membrane cultures that eventually differentiate to have an apical brush border, a characteristic phenotype of the normal small and large intestine [105]. Moreover, they also exhibit characteristic cell–cell adhesion properties similar to the intestine, including development of tight and adherens junctions [106,107]. HRV can infect both differentiated and undifferentiated Caco-2 cells, but requires trypsin during the entire course of infection for efficient replication [11]. Caco-2 cells also support HAstV replication, for which the usage of serum-free media during infection is recommended [41,108]. While Caco-2 cells express histo-blood group antigens, which are key attachment factors for HuNoV [109,110,111,112,113], they do not consistently support HuNoV replication in monolayer conditions [96,114].

HT-29 cells are a pluripotent, heterogeneous cell line that grows as a multilayer of unpolarized, undifferentiated cells with less than 5% differentiated mucus-secreting cells and columnar absorptive cells [115]. These cells have the potential to further differentiate: in the absence of glucose, HT-29 cells can undergo a typical enterocytic differentiation [116], whereas in the absence of serum, ~50% of cells differentiate into goblet-like cells expressing mucins [117]. HT-29 cells support in vitro growth of HRV [118], and a variant, the mucus-secreting HT-29-MTX line generated by differentiating HT-29 into mature goblet cells using methotrexate [115], has been successfully employed to investigate the role of glycans in promoting HRV virulence [14]. HT-29 cells can also support efficient replication of some HAstV serotypes when grown without glucose [41]. Unlike HRV and HAstV, HuNoV does not replicate in HT-29 cells [96,114,119].

HuNoV GI and GII strains have been previously cultivated in 3-dimensional models wherein intestinal epithelial cell line INT-407 was grown on collagen-coated porous microcarrier beads in rotating-wall vessel bioreactors [119], an approach also used for HuNoV replication with Caco-2 cells [120]. However, challenges in replicating this model have prevented its widespread implementation [121,122,123].

#### 3.1.2. B Cell Lines

After many unsuccessful attempts to grow HuNoV in immortalized cell lines, the identification of an immune cell tropism for a strain of murine norovirus (MNoV), a prominent small animal model for HuNoV studies, suggested potential utility in exploring immune cells for HuNoV cultivation [124]. GII.4 strains of HuNoV have since been cultivated in human-derived transformed B-cell line BJABs [29,77], an infection which is enhanced in the presence of histo-blood group antigen carbohydrates that facilitate viral attachment to the B cells [77]. However, low virus yield and inconsistent results among different laboratories have been reported [29].

#### 3.1.3. Non-Human Primate Cell Lines

African green monkey (*Cercopithecus aethiops*) kidney cell lines are the most common non-human primate cell lines used for enteric viral research. These cell lines are susceptible to many viruses due to the absence of type I interferon (IFN) and cyclin-dependent kinase inhibitor genes [125,126]. Indeed, African green monkey kidney cells were the first used for the growth of HRV [127].

Vero cells are the most widely accepted immortalized cell line for the development of human viral vaccines, and have been used for poliovirus, rabies virus, influenza virus, and HRV vaccine propagation [128], though yield of high-titer virus has been a frequent challenge. Recently, selective disruption of ten antiviral genes in Vero cells including neuraminidase-2 (NEU2) and RAD51 recombinase associated protein 1 (RAD51AP1) was shown to result in higher yields for HRV replication [129]. Although many unsuccessful attempts have been made to use Vero cells to propagate HuNoV [31,96,114], a recent study suggested that replication of HuNoV may be possible in the presence of trypsin and by disrupting six host genes in Vero cells, again including NEU2 and RAD51AP1 [30]. Validation by other groups will be important to demonstrate the widespread utility of Vero cells for HuNoV studies. Vero cells have also been found to support the growth of HAstV [41], though Caco-2 cells are generally more easily infected [130].

MA-104 cells were first reported as rhesus monkey kidney cells [131], but later karyological analysis determined that these originate from African green monkeys [132]. MA-104 cells have been the cell type of choice to cultivate HRV for many years [133,134], with well-documented protocols available [15]. Similar to Vero cells, MA-104s can support HAstV replication as well [41]. These cell lines are also used for the study of enteric RNA viruses causing extraintestinal disease including HAV and HEV [135,136,137].

##### Advantages and Disadvantages of Using Immortalized Cell Lines

Because they are cost-effective and have the potential to grow indefinitely, immortalized cell lines offer multiple advantages over alternate approaches (Table 2). They are easy to maintain and devoid of any ethical concerns associated with the usage of animals or human tissues. Although cell lines serve as a powerful tool for viral studies, due to the homogeneity of the cell population and the lack of host factors such as immune cells and the enteric nervous system, they do not necessarily represent the in vivo tissue environment. These lines have been genetically manipulated, either via transformation in cancer cell lines or via artificial expression of cancer genes to drive indefinite proliferation. This manipulation can alter key phenotypes including immune responses and cellular functions. Further, serial passaging can lead to genetic drift, driving heterogeneity in cell populations that can confer genotypic and phenotypic variation, both over time within a laboratory and between research groups [138].

### 3.2. Primary Cells

Primary cells are isolated directly from tissue and are grown in vitro, retaining morphological and functional properties of their tissue of origin. For viral studies, primary cells may facilitate adaptation of virus to in vitro conditions before transitioning to immortalized cell lines. For example, HRV is more efficiently grown in primary African green monkey kidney cells than immortalized lines [131,139], and multiple rounds of passaging in primary cells were critical to adapt virus for growth in MA-104 cells [15,139]. A potential link has been reported between RV infection and pancreatitis and subsequent autoimmune type-1 diabetes [140,141]; replication of HRV has also been demonstrated in primary monkey islet cells [142]. Human intestinal primary cells have also been recently utilized for replication of other enteric viruses such as HEV [143]. Moreover, HEV has also been cultivated successfully in extrahepatic primary cells such as hematopoietic cells, endometrial stromal cells and renal epithelium [144,145,146]. However, HuNoV has not been shown to replicate in primary cells [96].

#### Advantages and Disadvantages of Primary Cells

Primary cells offer advantages including maintenance of the physiological features and genetic makeup of the tissue of origin; they are also cost-effective in comparison to animal models. However, they also exhibit longer doubling times and limited growth potential compared to immortalized cells. They are difficult to maintain and may change with each passage, and cells taken from different sources can exhibit high levels of variation in responses to external stimuli [138] (Table 2).

### 3.3. Intestinal Enteroids

Because human enteric viruses can be challenging to cultivate in 2-dimensional cultures, especially in the case of HuNoV, a need for models that more accurately recapitulate human intestinal physiology arose. The differentiation of tissue-derived intestinal stem cells into 3-dimensional human intestinal enteroids (HIEs) using specific growth factors has provided a much-needed breakthrough for the study of human enteric viruses [147]. These structures resemble the in vivo human intestinal tissue architecture (hence they are also called mini-guts) in terms of having a columnar epithelium consisting of absorptive functional enterocytes and secretory lineages [147,148].

Clinical isolates of HRV from patient stool samples have been successfully cultivated in HIE cultures [18,20], with species specificity demonstrated by HRVs replicating much more efficiently than rhesus RVs [19]. Interestingly, HRV infection induces water influx into the HIE lumen, recapitulating HRV-induced diarrhea in vitro [19]. HIE cultures have been used to demonstrate the antiviral efficacy of type I, but not type III, IFN and nucleoside analog ribavirin against HRV, which varies by viral strain, supporting the use of these cultures towards personalized medicine approaches [149,150]. A variety of HuNoV strains have also been successfully cultivated in HIEs [20,31,151]. Studies exploring HuNoV pathogenesis in HIEs have revealed enterocytes to be a primary target for HuNoV replication, and indicated that cells derived from duodenum, jejunum, and ileum can be permissive to infection [31]. They have also identified viral strain-specific requirements for bile acids and histo-blood group antigens, regulated by FUT2 expression, for susceptibility of HIEs to HuNoV [16,31,152]. Importantly, genetic modifications of HIEs have been successfully performed using CRISPR-Cas9 approaches to explore FUT2 requirements and IFN regulation of HuNoV [32,33]; this capacity to genetically modify HIEs is likely to be increasingly applied to enteric viral cultivation studies (Figure 1). HIEs also permit efficient replication of HAstV, with HIEs derived from all intestinal segments supporting the growth of representative strains from all three clades [43,45,46]. These studies have shown that HAstV can infect multiple cell types such as goblet cells, mature enterocytes, and intestinal progenitor cells [43,45,46], and have revealed the importance of IFN-mediated antiviral responses against HAstV [45].

Because 3-dimensional enteroids have a closed structure, depending upon the pathogen being studied, it may be critical to explore whether luminal/apical entry requires derivation of a 2-dimensional monolayer, wherein the organoids are dissociated by enzymatic treatment and cells are seeded onto plates coated with matrigel [31,152,153], collagen mimicking the extracellular matrix [154], or polyethylene glycol [155]. Two-dimensional monolayers have the advantage of scaling up and can be used for high throughput screening studies [156]. Another option to provide simultaneous apical and basolateral access is to grow the 2-dimensional monolayer on a Transwell insert containing a porous membrane and coated with extracellular matrix-like proteins [156,157]. These Transwell inserts can also be used to co-culture immune and epithelial cells in the different chamber compartments, with the porous membrane permitting transport of secreted factors [158,159]. An alternate approach to provide apical access in a 3-dimensional enteroid system is to leverage a new technique of reverse enteroid development wherein manipulation of extracellular matrix proteins permits access to the apical surface [156]. These ‘api-cal-out’ enteroids can differentiate into various intestinal epithelial cell lineages, act as diffusion barriers and perform intestinal functions including nutrient absorption and mucus secretion [160], and have been recently applied to study the pathogenesis of pandemic viral strain SARS-CoV-2 [161,162], highlighting the potential for their application to other viral systems. Another consideration for these cultures is the absence of physical forces, such as fluid shear effect or peristaltic movement, characteristic of intestinal physiology. These forces regulate the behavior of intestinal cells [163] and their interaction with luminal contents [164]. Microfluidic devices for introducing shear fluid forces to generate a microenvironment similar to the gut, a model called “intestine-on-a-chip”, that employ monolayers derived from 3-dimensional organoids have thus been developed [165,166,167]. However, they require specialized technical expertise, microfabrication, and fluidic systems that are not universally accessible. These specialized systems, described in additional detail elsewhere [168], have been recently adapted for successful cultivation of HuNoV [169].

#### Advantages and Disadvantages of HIEs

Advantages of HIEs include improved recapitulation of intestinal physiology and potential for modification, though technically demanding, using genetic engineering tools. Moreover, once established, HIEs can be maintained on a long-term basis and potentially scaled up for genomic and drug screening. Most importantly, HIEs can be utilized to develop personalized medicine approaches since they are derived from genetically and phenotypically distinct individuals [170].

While this variation in HIEs depending on donor can be an advantage, it can also lead to challenges including high levels of variability in any given phenotype between cultures developed from different donors, as genetic background and factors such as age of the individuals can lead to distinct phenotypic responses [170]. HIE models generally lack components of the host microenvironment, as discussed above [171]. Finally, maintenance of HIEs is very expensive and time-consuming, requiring a substantial degree of expertise, compared to immortalized cell lines (Table 2).

## 4. In Vivo Tools to Study Human Enteric RNA Viruses

**Figure 2:**
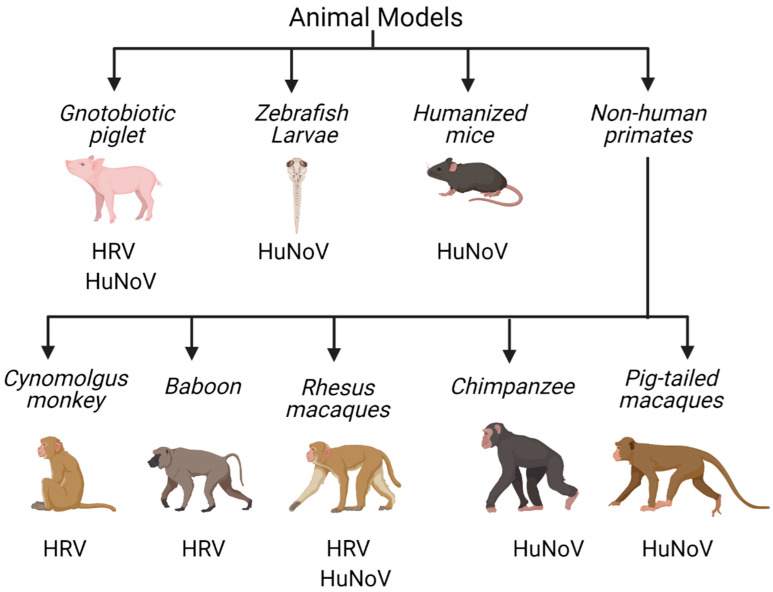
Animal models available for the study of HRV and HuNoV. Numerous animal models described to date support replication of HRV and HuNoV, including a variety of non-human primates as well as gnotobiotic pigs, zebrafish, and humanized mice. Created with BioRender.com (accessed on 21 January 2021).

While the study of viruses in vitro provides many key insights into virus–cell interactions, it is critical to complement these analyses with the study of infection in the context of an organism. Factors such as adaptive immune responses to and the influence of the microbiota on viral infection in the intestine can often be most readily achieved with the use of animal models [172,173] (Figure 2).

### 4.1. Non-Human Primates

Due to the genetic proximity of non-human primates (NHP), including vervet monkeys, cynomolgus monkeys, rhesus macaques, pig-tailed macaques, chimpanzees, and baboons, to humans, they expectedly share many anatomical, immunological, and physiological similarities. NHPs have served as important experimental models for enteric viral research, as they recapitulate the pathogenesis of infections in humans to a greater degree than other animal models [26,174,175,176]. Baboons and vervet monkeys infected with HRV exhibit viral shedding and elevated level of virus neutralizing antibodies [175], and cynomolgus monkeys similarly exhibit self-limiting diarrhea and shedding of infectious virus [26,27]. Chimpanzees have been used for HuNoV infections, wherein the duration of viral shedding and serum antibody responses in this model are similar to those in humans [40,177]. Pig-tailed macaques are also susceptible to HuNoV, exhibiting diarrheal illness, while rhesus macaques exhibit prolonged shedding and antibody responses in the absence of diarrheal symptoms [39,176].

Enteric viruses isolated directly from NHPs have also been used extensively for study of viral pathogenesis. For example, SA11 is a simian rotavirus initially isolated from a vervet monkey [178] which has subsequently been used as a model for the study of RV both in vivo and in vitro [179,180]. Study of another simian rotavirus strain, rhesus RV, contributed to the formulation of RV vaccines [181,182]. Recovirus is an enteric calicivirus isolated from rhesus macaques [183] which causes diarrhea in infected animals and can be used to study pathogenesis of and immunity to caliciviruses such as HuNoV in vivo [184]. In the last several years, AstVs have been increasingly identified in NHP samples, though these have not yet been extensively characterized [185,186].

### 4.2. Gnotobiotic Pigs

Gnotobiotic pigs, referring to pigs for whom the microbial status is well-defined and including germ-free status, have been a long-standing resource for the study of both the microbiota as well as enteric viruses due to their strong similarities to humans in pathophysiological responses [187]. Human microbiota samples can be efficiently transplanted into gnotobiotic piglets, resulting in microbial profiles similar to the donor samples [188].

Infection of gnotobiotic piglets with either HRV or HuNoV recapitulates human symptoms of acute viral gastroenteritis including diarrhea and fecal viral shedding, thus making these models extremely useful for immunological studies [25,189]. Gnotobiotic piglets have been extensively used for RV vaccine evaluation [190], and studies of HuNoV infection in gnotobiotic piglets demonstrate an association of HuNoV infection with intestinal epithelial cell apoptosis and barrier disruption, indicating a mechanism for HuNoV-induced diarrhea in humans [191]. Porcine enteric viruses, causing pathological symptoms of gastroenteritis such as diarrhea, vomiting, dehydration and even mortality in neonatal piglets, have been found which share similarity with HuNoV GII and HAstV strains [192,193,194].

### 4.3. Mouse Models

One of the most widely used animal models in biomedical sciences is the laboratory mouse (*Mus musculus*). Mice have many features that make them ideal for research studies, including a relatively fast reproduction rate with large litter sizes, the existence of large and well-controlled housing facilities in the majority of research institutions, and an enormous breadth of genetically modified models permitting interrogation of the sufficiency or necessity of factors for any given phenotype [195]. In the field of enteric virus research, mice can potentially be infected directly with human viruses. BALB/c and outbred Swiss SW55 mouse pups can be orally inoculated with HRV strains, with infection causing development of symptoms and histopathological changes associated with gastroenteritis [196,197]. Similarly, BALB/c *Rag2^−/−^Il2rg^−/−^* mice engrafted with human CD34+ hematopoietic stem cells have been used for intraperitoneal infection with HuNoV GII.4, wherein viral structural and nonstructural proteins were found to be expressed in the spleen and liver [35]. Interestingly, in this infection model the genetic background of the mice may play a more important role than the engraftment of human cells [35]. These models have not yet been widely used and may benefit from further development.

Genetically related mouse viruses are another option as models to further investigate the pathogenesis of enteric viruses in their natural hosts, which can then shed light on the characteristics of the related human virus as well. Murine rotavirus (mRV), first discovered fifty years ago, has been widely studied and causes diarrheal disease with particular severity in BALB/c mouse pups [198,199,200,201]. More recently, studies using mRV have revealed critical roles for IFNs in regulation of enteric viral infection [202,203], and identified roles for segmented filamentous bacteria as well as bacterial flagellin in mediating antiviral effects through accelerated epithelial cell turnover and IL-22/IL-18, respectively [204,205]. Similarly, murine norovirus (MNoV) [206], for which numerous phenotypically distinct viral strains have been described, has served as a powerful model for HuNoV since its discovery two decades ago. Numerous MNoV studies have contributed substantially to our understanding of the cellular and molecular mechanisms of NoV pathogenesis, revealing important regulatory aspects of the commensal microbiota, IFN signaling, and roles for nonstructural proteins in antagonism of host responses which are conserved with HuNoV [124,207,208,209]. A recent set of studies identified CD300LF as the host protein which serves as the MNoV receptor, and though human CD300LF does not mediate the same role for HuNoV, this finding raises intriguing questions about the potential existence of a proteinaceous receptor for HuNoV [124,210,211,212]. More recently, murine astrovirus (muAstV) was identified as a small animal model for HAstVs [213,214]. MuAstV has been shown to mediate viral interference against other enteric viruses in immunodeficient hosts [215], and its tropism for mucus-secreting goblet cells may also permit muAstV to regulate enteric bacterial pathogens [46,216]. Although mouse models of viral infection may not reflect all clinical outcomes of human enteric viruses, their importance in interrogating and defining key aspects of viral regulation in vivo cannot be denied.

### 4.4. Zebrafish

Recently, zebrafish (*Danio rerio*) have gained prominence as in vitro and in vivo models for the study of viral pathogenesis. Zebrafish offer numerous advantages including being inexpensive, breeding rapidly, and being genetically tractable [217,218]. In addition, zebrafish are transparent during development, facilitating visualization of internal structures and viral infection. This model shares genetic and physiologic similarities to humans including innate and adaptive immunity, making them widely used vertebrate models of human diseases. Recently, zebrafish larvae have been used as a robust replication model for HuNoV. GI and GII viruses replicate to high titers, with virus detectable in both intestinal and hematopoietic tissues, consistent with the possible dual tropism for HuNoV [34]. In addition, an increase in expression of several immune genes was also observed [34]. The zebrafish model has also been utilized for high throughput anti-HuNoV drug screening [219,220]. Of interest, a transgenic zebrafish expressing green fluorescent protein under the influence of an IFN stimulated gene promoter has been developed, providing an in vivo tracking system for viral infections [221].

### 4.5. Turkeys

Turkeys have proven to be useful small animal models for the study of AstV pathogenesis and immunity [222,223]. Turkey poults infected with turkey AstV (TAstV-2) show clinical symptoms including age-dependent diarrhea, and have been used to identify the viral capsid as a critical mediator of diarrhea and to test therapeutic antiviral strategies against AstVs [224,225].

## 5. Conclusions and Future Directions

Viral gastroenteritis remains a serious global health concern, as we currently lack vaccines for HuNoV and HAstV, and the efficacy of the RV vaccine may be limited in some countries due to unclear environmental factors. While the lack of suitable in vitro and in vivo model systems that could reliably recapitulate the primary aspects of viral pathogenesis in humans has been a limitation, recent advances in cultivation systems in human enteroids as well as the expansion of available animal models offer promise for future breakthroughs.

A current challenge with most current in vitro systems is the absence of host factors such as immune cells and the microbiota; these are areas of ongoing development [158,226,227]. In addition, improved immortalized cell line options for some of the human viruses would facilitate application of drug or genetic screening approaches. Immortalization of those cells that are routinely infected in humans in vivo, or overexpression of the viral receptor in already established and easy-to-use lines, could yield these resources which would be a great boon to the field.

In vivo models naturally provide the most clinically relevant information about mechanisms of pathogenesis or host–virus interactions. The models discussed here have provided extremely useful insights, but improvements are always possible. Robust and reproducible systems to reliably infect inexpensive and manipulatable small animal models such as mice and zebrafish with human enteric viruses would be tremendously useful and may rely on generation of transgenic animals expressing human viral receptors, similar to the introduction of the poliovirus receptor into mice [228]. Additional future advances in these model systems will almost certainly continue to yield an improved understanding of the pathogenesis of these enteric viruses, which will be key to improved vaccine and therapeutic approaches to combat the global burden of acute viral gastroenteritis.

## Figures and Tables

**Table 1:** Summary of commonly employed in vitro and in vivo models to study human rotavirus, norovirus, and astrovirus, with most broadly-used approaches shown in red.

Virus	Method	Origin	Experimental Model	Viral Strain(s)	References
*Human* *Rotavirus*	*in vitro*		*Immortalized cells*		
		Human colon adenocarcinoma	Caco-2	Trypsin-activated HRV-Wa or SA114F	[11,12]
		Human colorectal adenocarcinoma	HT-29	Trypsin-activated SA114F	[12]
		Human colorectal adenocarcinoma	Genetically-modified HT-29 (*STAG2^−/−^*, *STAT1^−/−^*, and *STING^−/−^*) HT-29	Trypsin-activated HRV-Wa, G2P[4], G4P[6], G12P[4], G9P[8], G8P[11], G8P[10]	[13]
		Human colorectal adenocarcinoma	HT29-MTX	Trypsin-activated SA114F	[14]
		African Green Monkey Kidney	MA-104 and Vero	Trypsin-activated HRV	[15]
		African Green Monkey Kidney	Genetically-modified (*LRGUK*^−/−^, *WR62^−/−^*, *EMX2^−/−^*) Vero cells	Trypsin-activated RV3, CDC-9. Rotarix and 116E	[16,17]
			*Human Intestinal Enteroids (HIEs)*		
		Human intestinal biopsies	3D-HIEs	Trypsin activated G1P[8] and G9P[8]	[18,19]
		Human intestinal biopsies	Differentiated 2D-monolayer and Transwell HIEs	Trypsin activated HRV	[20]
		Human intestinal biopsies	2D-Monolayer and transwell	Trypsin activated HRV G3P[8]	[21]
	*in vivo*		*Animal Model*		
		Rodent	Guinea pig	HRV-Wa	[22,23]
		Pig	Gnotobiotic piglet	HRV-Wa	[24,25]
		Non-human primate	Cynomolgus monkeys	HRV-Wa or infected stool filtrate	[26,27,28]
*Human Norovirus*	*in vitro*		*Immortalized Cell lines*		
		Human malignant B-cells	BJAB (with or without HT-29 co-culture)	GII.4 stool filtrate	[29]
		African Green Monkey Kidney	Vero	Trypsin-activated GII.3 & GII.4 stool filtrate	[30]
			*HIEs*		
		Human intestinal biopsies	Differentiated 2D-monolayer & Transwell HIEs	Bile-treated GI.I, GII.3 & GII.4 stool filtrate	[20,31]
		Human intestinal biopsies	Genetically-modified (*IFNAR1*^−/−^, *IFNLR1*^−/−^, *STAT1^−/−^, MAVS*^−/−^, and *STAT1^−/−^STAT2*^−/−^) HIEs	GII.3 & GII.4 stool filtrate	[32]
		Human intestinal biopsies	Genetically-modified (*FUT2*^−/−^) HIEs	GII.3, GII.4 & GII.17 stool filtrate	[33]
	*in vivo*		*Animal Model*		
		Fish	Zebrafish	GI.7, GII.2, GII.3, GII.4, GII.6 from stool suspension	[34]
		Rodent	Humanized BALB/c *Rag^−/−^Il2rg^−/−^* mice (engrafted with human CD34+)	GI.3a, GII.4, GII.6 from stool suspension	[35]
		Pig	Gnotobiotic piglets	GII.4 stool filtrate	[36,37]
		Pig	*Rag2^−/−^Il2rg^−/−^* gnotobiotic piglets	GII.4 stool filtrate	[38]
		Non-human primate	Pig-tailed macaques	GII.3 from stool suspension	[39]
		Non-human primate	Chimpanzee	GI.1 stool filtrate	[40]
*Human Astrovirus*	*in vitro*		*Immortalized Cell line*		
		Human colon adenocarcinoma	Caco-2	HAstV-1, 2, 3, 4, 5, 6, 7	[41,42,43]
		Human colon adenocarcinoma	Genetically-modified Caco-2 (*CLTC* silencing)	Trypsin-activated HAstV-8	[44]
		Human colorectal adenocarcinoma	HT-29	Trypsin-activated HAstV-1, 2, 3, 4, 5, 6, 7	[41]
			*HIEs*		
		Human intestinal biopsies	3D HIEs	HAstV-1	[45,46]
		Human intestinal biopsies	Differentiated 2D-monolayer in transwell	HAstV-VA1; HAstV-MLB1 & Trypsin- activated HAstV-1	[43,47]

**Table 2:** Considerations in selection of method(s) for the study the human RNA enteric viruses.

Experimental Considerations	Immortalized Cell Lines	Primary Cells	HIEs	Animal Model
**Expense**	Low	Moderate	High	High
**Maintenance demands**	Low	Moderate	Moderate to high	High
**Biological relevance**	Low	High	High	High
**Reproducibility**	High	Moderate	Moderate	Moderate (within institutions)
**Genetic manipulation**	Easy	Difficult	Moderate	Difficult
